# Role of religion and spirituality in medical patients: Confirmatory results with the SpREUK questionnaire

**DOI:** 10.1186/1477-7525-3-10

**Published:** 2005-02-10

**Authors:** Arndt Büssing, Thomas Ostermann, Peter F Matthiessen

**Affiliations:** 1 Krebsforschung Herdecke, Department of Applied Immunology and Cancer Service, Herdecke Community Hospital, Gerhard-Kienle-Weg 4, 58313 Herdecke, Germany; 2 Department of Medical Theory and Complementary Medicine, University Witten/Herdecke, Gerhard-Kienle-Weg 4, 58313 Herdecke, Germany

**Keywords:** Questionnaires, Religion and Medicine, Spirituality and Religion, coping, chronic disease, cancer

## Abstract

**Background:**

Spirituality has become a subject of interest in health care as it is was recognized to have the potential to prevent, heal or cope with illness. There is less doubt that values and goals are important contributors to life satisfaction, physical and psychological health, and that goals are what gives meaning and purpose to people's lives. However, there is as yet but limited understanding of how patients themselves view the impact of spirituality on their health and well-being, and whether they are convinced that their illness may have "meaning" to them. To raise these questions and to more precisely survey the basic attitudes of patients with severe diseases towards spirituality/religiosity (SpR) and their adjustment to their illness, we developed the SpREUK questionnaire.

**Methods:**

In order to re-validate our previously described SpREUK instrument, reliability and factor analysis of the new inventory (Version 1.1) were performed according to the standard procedures. The test sample contained 257 German subjects (53.3 ± 13.4 years) with cancer (51%), multiple sclerosis (24%), other chronic diseases (16%) and patients with acute diseases (7%).

**Results:**

As some items of the SpREUK construct require a positive attitude towards SpR, these items (item pool 2) were separated from the others (item pool 1). The reliability of the 15-item the construct derived from the item pool 1 respectively the 14-item construct which refers to the item pool 2 both had a good quality (Cronbach's alpha = 0.9065 resp. 0.9525). Factor analysis of item pool 1 resulted in a 3-factor solution (i.e. the 6-item sub-scale 1: "Search for meaningful support"; the 6-item sub-scale 2: "Positive interpretation of disease"; and the 3-item sub-scale 3: "Trust in external guidance") which explains 53.8% of variance. Factor analysis of item pool 2 pointed to a 2-factor solution (i.e. the 10-item sub-scale 4: "Support in relations with the External life through SpR" and the 4-item sub-scale 5: "Support of the Internality through SpR") which explains 58.8% of variance. Generally, women had significantly higher SpREUK scores than male patients. Univariate variance analyses revealed significant associations between the sub-scales and SpR attitude and the educational level.

**Conclusions:**

The current re-evaluation of the SpREUK 1.1 questionnaire indicates that it is a reliable, valid measure of distinct topics of SpR that may be especially useful of assessing the role of SpR in health related research. The instrument appears to be a good choice for assessing a patients interest in spiritual concerns which is not biased for or against a particular religious commitment. Moreover it addresses the topic of "positive reinterpretation of disease" which seems to be of outstanding importance for patients with life-changing diseases.

## Background

Spirituality has become a subject of interest in health care, and an increasing number of studies, commentaries and reviews examine the connection between religiosity/spirituality and health, its potential to prevent, heal or cope with diseases [1-10]. Moreover, research has confirmed that spiritual well-being is positively associated with quality of life, fighting-spirit, but also fatalism, yet negatively correlated with helplessness/hopelessness, anxious preoccupation, and cognitive avoidance [11]. Indeed, there is evidence that spirituality is important in coping with illness, as spiritual well-being offers some protection against hopelessness and despair in terminally ill patients [12-16].

However, although religiosity and spirituality were interchangeable words, these constructs may not be identical. It is well established to divide Religiosity into three sub-constructs: Intrinsic, Extrinsic, and Quest Religiosity [17-20], while the construct Spirituality was divided into the following sub-constructs: Cognitive Orientation Towards Spirituality, Experiential/Phenomenological Dimension of Spirituality, Existential Well-Being, Paranormal Beliefs, and Religiousness [21].

The measurability and operability of spirituality and religiosity remains a problem and thus several questionnaires address this topic. Most of them measure beliefs of specific religious groups, and ask about the relationship with God (i.e. the *Spiritual Well-Being Scale *[22], the *Daily Spiritual Experience Scale *[23], or the *Santa Clara Strength of Religious Faith Questionnaire *[24], while only a few took into account that several patients are offended by institutional religion, but may have an interest in distinct forms of spirituality, respectively in a more personal search for spiritual fulfilment [25,26]. The *Functional Assessment of Chronic Illness Therapy – Spiritual Well-Being *(FACIT-Sp) scale has a much more open design [27], but, however, the 12 items of this instrument which made up 2 main factors (labelled "Meaning/Peace" and "Faith") may not really meet the situation of patients with severe and life-threatening diseases. In the post-treatment orientation phase of cancer patients, more existentialistic issues in the patients' attempt to manage the implications of their disease in daily life are of outstanding importance [28,29] The same is true for hospitalised cancer patients [30].

There is less doubt that values and goals are important contributors to life satisfaction, physical and psychological health, and that goals are what gives meaning and purpose to people's lives [31-33]. Moreover, health can be conceptualized as a competence to gain control for the design of the biography [34]. But in face of a life-threatening diseases, do patients find meaning and purpose in their life? Many of them rely on religious beliefs to relieve stress, retain a sense of control, maintain hope and their sense of meaning and purpose in life [35], while others may lose faith in their religious beliefs, and seek for alternatives [28,29]. There is as yet but limited understanding of how patients themselves view the impact of spirituality on their health and well-being, and whether they are convinced that spirituality may offer some beneficial effects.

To raise these questions and to more precisely survey the basic attitudes of those patients towards spirituality/religiosity (SpR) and their adjustment to their illness, we developed the SpREUK questionnaire [28,29,36-39]. We defined the multi-dimensional construct "Spirituality" as an "individual and open approach in the search for meaning and purpose in life, as a search for transcendental truth which may include a sense of connectedness with others, nature, and/or the divine" [28]. The main sub-scales of our instrument may thus correspond to MacDonald's spirituality constructs of an "Existential Well-Being" [21] which describes a meaning and purpose for existence, and the perception of self as being competent and able to cope with the difficulties of life and limitations of human existence, and to the construct of an "Cognitive Orientation Towards Spirituality" which is identified by beliefs, attitudes, and perceptions regarding the nature and significance of spirituality, as well as having relevance and importance for personal functioning.

In this article we report the re-validation of the SpREUK 1.1 questionnaire (SpREUK is an acronym of the German translation of "Spiritual and Religious Attitudes in Dealing with Illness"), an instrument designed to examine attitudes of patients with life-threatening and chronic diseases towards spirituality/religiosity.

## Methods

### Procedure and subjects

All individuals were informed of the purpose of the study, were assured of confidentiality, and gave informed consent to participate. The patients were recruited consecutively in the cancer service, the multiple sclerosis service, and two internal medical units of the Communal Hospital in Herdecke (Germany). All subjects completed the questionnaire by themselves. Demographic information is provided in Table 1.

**Table 1:** Demographic data and SpREUK scores of 257 subjects

	**%**	**Search Meaning **(50.6 ± 25.9)	**Message Disease **(70.4 ± 20.9)	**Trust Guidance **(70.0 ± 26.5)	**Support External **(59.0 ± 23.9)	**Support Internal **(62.3 ± 24.3)
**sex**		**	**	*	**	*
female	70	54.7 ± 26.0	73.4 ± 20.3	72.9 ± 24.4	61.8 ± 23.1	64.9 ± 22.6
male	30	**40.8 ± 22.9**	63.4 ± 20.8	63.0 ± 30.0	52.4 ± 24.5	55.6 ± 27.1

**age**		(*)		**	*	*
< 30 years	3	**31.8 ± 19.8**	60.9 ± 24.6	**40.6 ± 20.5**	**38.0 ± 18.1**	**43.0 ± 20.7**
30–49 years	38	49.7 ± 26.2	70.4 ± 20.2	63.4 ± 26.9	55.3 ± 23.4	58.8 ± 23.9
50–69 years	45	54.2 ± 26.2	72.2 ± 19.6	74.7 ± 24.9	62.5 ± 22.4	65.8 ± 23.2
> 70 years	12	46.2 ± 23.6	67.7 ± 25.7	**79.8 ± 23.5**	63.4 ± 28.2	64.5 ± 28.0

**marital status**		*		*	*	
married	65	47.2 ± 26.0	69.3 ± 21.3	70.1 ± 27.0	56.1 ± 24.6	60.5 ± 26.0
living with partner	11	52.5 ± 20.1	73.3 ± 21.3	**57.1 ± 24.7**	58.3 ± 16.8	63.2 ± 13.9
divorced	9	**65.0 ± 26.9**	75.4 ± 19.3	74.1 ± 25.8	**69.9 ± 19.7**	68.5 ± 24.7
alone	10	55.9 ± 29.6	71.3 ± 20.4	72.3 ± 25.8	61.6 ± 27.2	65.9 ± 23.7
widowed	5	51.4 ± 17.8	68.2 ± 19.6	**84.3 ± 17.8**	**73.5 ± 18.2**	62.5 ± 22.9

**education**^1^		**	**		**	**
level 1	25	**36.6 ± 24.9**	**58.6 ± 20.4**	69.0 ± 26.1	55.0 ± 24.1	58.9 ± 24.6
level 2	29	44.4 ± 27.8	67.8 ± 19.5	68.9 ± 29.6	50.6 ± 25.7	53.5 ± 29.2
level 3	37	**65.5 ± 24.7**	76.9 ± 16.7	75.8 ± 19.8	**68.2 ± 19.2**	69.1 ± 15.3
other	9	**65.5 ± 24.94**	74.6 ± 21.6	74.2 ± 21.2	**67.9 ± 19.8**	**75.0 ± 19.0**

**disease**		**	**	**	**	**
Cancer	51	55.4 ± 24.6	73.8 ± 19.7	74.2 ± 23.4	62.3 ± 22.5	64.3 ± 22.3
Multiple Sclerosis	24	**35.8 ± 22.5**	**59.0 ± 19.1**	**56.8 ± 28.0**	**48,0 ± 23.3**	**52.9 ± 25.8**
Chronic diseases	16	**56.5 ± 25.7**	75.0 ± 19.3	69.8 ± 27.5	63.1 ± 26.1	68.6 ± 26.1
Acute diseases	7	44.7 ± 26.4	74.8 ± 27.1	76.4 ± 30.6	60.6 ± 23.9	67.6 ± 23.9

**duration of disease**						
< 0.5 years	19	48.2 ± 26.1	73.3 ± 18.6	68.8 ± 27.7	58.4 ± 21.7	63.3 ± 20.2
0.5–1 years	12	53.1 ± 24.9	79.0 ± 19.7	67.9 ± 28.6	58.1 ± 20.6	58.0 ± 26.0
1–3 years	26	54.1 ± 24.4	72.5 ± 20.2	71.1 ± 23.1	60.6 ± 22.9	63.7 ± 22.9
3–5 years	12	46.8 ± 27.6	61.9 ± 19.4	66.4 ± 28.2	56.7 ± 26.1	56.7 ± 28.3
> 5 years	31	47.3 ± 26.4	70.8 ± 22.1	68.0 ± 28.1	56.7 ± 27.0	61.9 ± 26.0

**confession**		**		**	**	**
Christian	80	52.9 ± 25.4	71.2 ± 20.8	76.0 ± 21.3	62.2 ± 22.8	63.8 ± 24.3
Others	3	**58.3 ± 18.0**	70.8 ± 17.1	**85.4 ± 15.7**	**65.7 ± 23.5**	**83.0 ± 16.8**
None	17	**38.7 ± 26.9**	67.0 ± 22.2	**38.2 ± 28.3**	**41.7 ± 22.8**	**50.9 ± 22.4**

**Spiritual attitude**		**	**	**	**	**
R+S+	32	**71.1 ± 20.2**	77.9 ± 18.5	**85.4 ± 15.0**	**75.7 ± 14.0**	**74.6 ± 19.9**
R+S-	36	**42.1 ± 21.0**	68.2 ± 19.8	**81.3 ± 16.6**	59.1 ± 20.3	61.8 ± 22.2
R-S+	9	**66.8 ± 14.7**	76.7 ± 22.0	**50.2 ± 15.6**	63.8 ± 20.6	67.9 ± 16.7
R-S-	23	**29.0 ± 17.6**	61.1 ± 21.3	**37.8 ± 22.6**	**33.3 ± 19.0**	**42.9 ± 23.6**

^1^Increasing educational level (based on German school system): 1 = secondary education (Hauptschule), 2 = secondary education (junior high; Realschule), 3 = high school education (Gymnasium). Scores are significantly different (** p < 0.01; * p < 0.05; (*) 0.05 < p < 0.10; Kruskal-Wallis-Test for asymptomatic significance).

Deviations of >15% from the mean were highlighted.

The sample contained 257 subjects of whom 70% were women. The mean age was 53.3 ± 13.4 years. The majority had a Christian nomination (80%), 17% had no religious orientation, and 3% other nominations. Cancer was diagnosed in 51%, multiple sclerosis in 24%, and other chronic diseases in 16% (i.e. Hepatitis C, liver cirrhosis, inflammatory bowel disease, severe hypertension etc.); 7% of the individuals were patients with acute diseases (i.e. prolapsed intervertebral disc, stomach ulcer, heart arrhythmia etc). Patients in final stages of their disease were not enrolled.

### Measures

The items of the SpREUK 1.0 were developed with the patients' input (cancer service of the Herdecke Community Hospital) and experts' statements (physicians, priest and chaplains working with patients) [28,36], rather than from theoretical concepts. Nevertheless, the original SpREUK 1.0 questionnaire heeded the concept of "internal resp. external locus of control" by Rotter [40] and Levenson [41], "passive, active or collaborative religious coping" by Pargament [41], and the search for "meaning in life" described by Emmons [32,33]. In the final step of the questionnaire design, the items were improved with respect to already existing questionnaires dealing with the topics of religion and spirituality in patients care [36].

According to a previously conducted reliability and factor analysis [36,37] the SpREUK 1.0 version had the following scales: (1) Search for meaningful support, (2) Guidance, control and message of disease, (A) Support in relations with the external through spirituality/religiosity, and (B) Stabilizing the inner condition through spirituality/religiosity.

In order to more precisely differentiate the three topics guidance, control and message of disease in scale 2 of the version 1.0, for the current version of the questionnaire, six new items were added (i.e. F3.5: "My illness is a chance for my own development."; F3.7: "Because of my illness, I reflect on what is essential in my life"). All items were scored on a 5-point scale from disagreement to agreement (0 – does not apply at all; 1 – does not truly apply; 2 – don't know; 3 – applies quite a bit; 4 – applies very much). The SpREUK scores are referred to a 100% level (4 "applied very much" = 100%).

### Statistical analysis

Reliability and factor analysis of the new inventory were performed according to the standard procedures. Next, to combine several items with similar content, we relied on the technique of factor analysis which examines the correlations among a set of variables, and to achieve a set of more general "factors." Factor analyses were repeated rotating different numbers of items in order to arrive at the solution which demonstrated both the best simple structure and the most coherence.

Differences in the SpREUK scores were tested using the Kruskal-Wallis-Test for asymptomatic significance. We judged p < 0.05 significant, and 0,05 < p < 0.10 as a trend.

To tested the impact of several variables on the SpREUK sub-scales, we performed analysis of univariate variance (ANOVA). As in several cases Levene's test for equality of variances was significant, and we judged p < 0.01 as significant.

All statistical analyses were performed with SPSS for Windows 10.0.

## Results

### Reliability

In order to eliminate items from the item pool that were not contributing to the questionnaire reliability, the reliability of the scale and distinct sub-scales was evaluated with internal consistency coefficients, which reflect the degree to which all items on a particular scale measure a single (unidimensional) concept.

Our item pool consisted of the previously established set of items [28,36,37] and 6 new items which were added to differentiate the topic "Guidance, control and message of disease" of the version 1.0. As some of the questions require a positive attitude towards SpR, these items (item pool 2) were separated from the others (item pool 1).

Reliability analysis revealed that 6 items from the new item pool 1 had a poor corrected item-total correlation and thus were eliminated (however, several from the previous "Locus of Control" topic): F1.2 ("I do not need spiritual advice, I know by myself what should be done"; 0.033), F1.3 ("Spiritual/religious ideas are out-of-date"; 0.091), F2.1 ("I have no influence on my life, it is fixed by fate"; 0,025), F2.2 ("I accept my illness and bear it calmly"; 0.073). F2.3 ("My doctor or therapist helps me to keep my illness at bay"; 0.037), and F3.1 ("Whatever happens, I have trust in my inner strength"; 0.173). One item (F3.6 "The "true being" ("inner core") can not be affected by illness") was omitted because of a weak reliability (0.2997) and – even more important – it points to a distinct "field of meaning" that would need more items in the questionnaire, and thus will be used as marker item until the construct will be revised for this topic.

As shown in Table 2, the 15-item construct derived from the item pool 1 had a good quality (Cronbach's alpha = 0.9065). The 14-item construct which refers to the item pool 2 (which is identical to the old item pool 2 as described in [36]) had a very good quality (Cronbach's alpha = 0.9525).

**Table 2:** Mean values of the items from SpREUK 1.1 and reliability analysis

	Factors and Items	Mean value (Score 0–4)	Standard deviation	loading	corrected Item-Total correlation	Alpha if Item deleted (α = 0.9065)
	**1: Search for meaningful support**					
1.5	finding access to a spiritual source can have a positive influence on illness	2.21	1.30	.776	.7597	.8940
1.1	Spiritual attitude	1.96	1.37	.733	.6231	.8994
1.6	searching for an access to SpR	1.81	1.38	.730	.7641	.8935
1.9	urged to spiritual/religious insight	1.99	1.32	.721	.7593	.8940
1.7	others might teach and help to develop spirituality	2.13	1.31	.704	.6881	.8970
1.4	illness has brought renewed interest in SpR questions	1.97	1.40	.636	.6054	.9002
	**2: Positive interpretation of disease**					
3.5	illness as a chance for development	2.55	1.30	.813	.7135	.8959
3.4	illness has meaning	2.40	1.33	.703	.6901	.8968
3.2	illness as a hint to change life	2.86	1.05	.604	.6061	.9006
3.7	reflect on what is essential in life because of the illness	3.35	0.77	.570	.2975	.9085
2.4	able to affect the course of illness by themselves	2.58	1.18	.526	.4067	.9066
3.3	illness encourages me to get to know myself better	2.98	1.05	.457	.5026	.9037
	**3: Trust in external guidance**					
2.6	Religious attitude	2.71	1.24	.846	.5087	.9033
2.5	trust in a higher power.	2.71	1.24	.810	.5327	.9028
1.8	looking for purpose and meaning in life	3.02	1.21	.295	.4005	.9072

	Factors and Items	Mean value (Score 0–4)	Standard deviation	loading	corrected Item-Total correlation	Alpha if Item deleted (α = 0.9525)

	**4: Support in relations with the External life through SpR**					
4.1	plays a major role in life	2.23	1.38	.819	.7934	.9479
4.3	helps to manage life more consciously	2.64	1.18	.814	.9187	.9452
4.2	provides deeper connection with the world around	2.52	1.20	.807	.82112	.9475
4.4	helps to cope better with illness	2.49	1.23	.806	.8718	.9463
4.7	helps to restore mental and physical health	2.30	1.17	.720	.8530	.9465
4.8	practicing with others deepens SpR	1.80	1.34	.663	.6189	.9523
4.6	helps to view disease as a beneficial challenge for own development	2.06	1.24	.657	.8130	.9474
4.9	practicing alone and in silence deepens SpR	2.56	1.21	.594	.6139	.9523
4.10	distinct places stimulate SpR	2.64	1.32	.584	.5709	.9537
4.5	People who share SpR attitudes are important	2.47	1.17	.385	.7956	.9479
	**5: Support of the Internal life through SpR**					
5.4	refers to an inner power	2.13	1.27	.765	.4374	.9572
5.1	provides feeling of contentment and inner peace	2.64	1.19	.732	.8671	.9465
5.2	promotes inner strength.	2.46	1.21	.713	.8757	.9462
5.3	refers to a higher (external) power	2.74	1.30	.574	.7470	.9491

Thus, the internal consistency of the 29-item SpREUK 1.1 construct was sufficiently high. The level of difficulty (LoD = 2.482 [mean value] / 4) is 0.6205 for item pool 1 resp. (LoD = 2.406 [mean value] / 4) 0.6014 for item pool 2. With the exception of item F3.7 ("I reflect on what is essential in my life because of the illness"; LoD = 0.838), all values are in the acceptable range from 0.2 to 0.8.

### Factor analysis

To combine several items with similar content, we relied on the technique of factor analysis which examines the correlations among a set of variables, and to achieve a set of more general "factors." Factor analyses were repeated rotating different numbers of items in order to arrive at the solution which demonstrated both the best simple structure and the most coherence.

With a Kaiser-Mayer-Olkin value of 0.850 (item pool 1) resp. 0.939 (item pool 2), which measures the degree of common variance, the 15 resp. 14-item-pool seems to be suitable. Barlett's test for non-sphericity was highly significant (p < 0,001).

Primary factor analysis of item pool 1 pointed to a 5-factor solution. However, due to a low item number in the tentative subscales 2–5 (with 2 or 3 items each), we favoured the more appropriate 3-factor solution which explains 53.8% of variance (Table 2). Sub-scale 1 ("Search for meaningful support") with its 6 items had a Cronbach's alpha of 0.8549, sub-scale 2 ("positive interpretation of disease ") with its 6 items had an alpha of 0.8000, while the 3-item sub-scale 3 ("trust in external guidance") had an alpha of 0.6625. An spiritual attitude loads to sub-scale 1, while a religious orientation loads to sub-scale 3.

Factor analysis of item pool 2 (Table 2) pointed to a 2-factor solution which explains 58.8% of variance. The 10-item subs-scale 4 ("Support in relations with the External life through SpR") had a Cronbach's alpha of 0.9400, while sub-scale 5 ("Support of the Internality through SpR") with its 4 items had an alpha of 0.7828.

Thus, this the internal consistency of the item pool 1 was sufficiently high. However, there are several inter-correlations between the sub-scales (Table 3). "Search for meaningful support" correlated negatively with "trust in external guidance" and slightly with the "positive interpretation of disease". Moreover, "trust in external guidance" negatively correlated with "positive interpretation of disease".

**Table 3:** Component Transformation Matrix

**Scale**	**Search Meaning**	**Message Disease**	**Trust Guidance**	**Support External**	**Support Internal**
**1 Search Meaning**	**.745**	.566	.352		
**2 Message Disease**	-.304	**.758**	-.577		
**3 Trust Guidance**	-.594	.323	**.737**		
**4 Support External**				**.861**	-.509
**5 Support Internal**				-.509	**.861**

Components 1, 2 and 3 explain 53.8% of variance, while components 4 and 5 explain 58.8% of variance.

Analysis of the "side-loadings" of item pool 1 (only values > 0.35 were take into account) reveal that items F1.8 ("looking for purpose and meaning in life") load good on sub-scale 2 (0.414). Analysis of the side-loadings of item pool 2 revealed that several items load also on the other sub-scale. Moreover, sub-scale 4 showed a strong but negative inter-correlation with sub-scale 5 (Table 3).

### Relation between SpREUK scores and demographic variables

The highest scores were found for the sub-scales 2 and 3 ("positive interpretation of disease" resp. "trust in external guidance"), the lowest for sub-scale 1 ("Search for meaningful support"). Means and standard deviations for study variables are provided in Table 1.

Women had significantly higher SpREUK scores than male patients. With respect to age, the lowest SpREUK scores were found in the group of < 30 years of age. With increasing age, the trust in a higher supporting presence (sub-scale 3) and the beneficial effects of resp. support through SpR increased.

With respect to the marriage status, widowed patients obviously has to rely on external guidance (sub-scale 3) but not the patients living with a partner not married with. Widowed and divorced patients find support in external relations through their SpR engagement (sub-scale 4), while – in contrast to married patients which may find hold in their partnership – especially divorced patients are in search for meaningful support (sub-scale 1).

Search for meaningful support and positive interpretation of diseases were depending on the educational level, as patients with lower educational level had significantly lower scores than those with a higher level. A higher educational level was associated with higher scores in the sub-scales 4 and 5 which deals with the beneficial effects of SpR.

Illness itself (but not the duration of disease) has a significant impact on the SpREUK scores, as MS patients had the lowest scores in all 5 sub-scales. The SpREUK scores of cancer patients revealed slight differences when compared to patients with other chronic diseases.

With the exception of sub-scale 2, patients without confessional affiliations had the lowest scores for all sub-scales, indicating that the "message of disease" was not depending on a denomination. Surprisingly, the few patients with other than a Christian orientation had the highest scores for sub-scales 1, 3, 4, 5.

Since nominational affiliation is not necessarily identical with religiosity or spirituality, we asked whether the patients would describe themselves as religious or spiritual [28,34,35]. Thirty-two % reported themselves as both religious and spiritual **(R+S+)**; 35% as religious, but not spiritual **(R+S-)**; 23% as neither religious nor spiritual **(R-S-)**; 10% claimed that they were spiritual, but not religious **(R-S+)**. Thus, the numbers of patients with denominational affiliation and self-reported spiritual/religious attitudes is somewhat similar. A spiritual attitude (R+S+ and R-S+) was associated with "search for meaningful support" and "positive interpretation of disease", while a religious attitude (R+S+ and R+S-) was associated with the highest scores for the "trust in external guidance" sub-scale 3.

The living area and the duration of diseases had no significant impact on the SpREUK scores.

### Correlation with SpR practice

As shown in Table 4, there were moderate to strong correlations between the SpREUK sub-scales and the engagement in a SpR practice as measured by the SpREUK-P manual [28,29]. The new version of SpREUK-P [29] measures (1) conventional religious practice (praying, church attendance etc.), (2) nature-oriented practice (healing effect on environment etc.), (3) existentialistic practice (self-realization, spiritual development, higher level of consciousness etc.), (4) unconventional spiritual practice (meditation, rituals, body-mind discipline etc.), and (5) humanistic practice (make an effort for other people etc.). The "nature-oriented practice", "humanistic practice" and the "unconventional spiritual practice" were only weakly associated with "Trust in external guidance". In contrast, a "conventíonal religious practice" was strongly correlated with "Search for meaningful support", "Trust in external guidance" and both "Support through SpR" scales 4 and 5. An "unconventional spiritual practice" was associated more with "Search for meaningful support" and " Support in relations with the External life through SpR", while an "esistentialistic practice" was associated stronger with "positive interpretation of disease" and "Support in relations with the External life through SpR.

**Table 4:** Pearson correlation between SpREUK sub-scales and SpR practice^1^

	**Search Meaning**	**Message Disease**	**Trust Guidance**	**Support External**	**Support Internal**
**SpREUK-P engagement scores**					
conventional religious practice	.577 **	.424 **	.642 **	.691 **	.624**
nature-oriented practice	.247 **	.246 **	.266 *	.358 **	.334 **
existentialistic practice	.437 **	.530 **	.411 **	.479 **	.439 **
unconventional spiritual practice	.498 **	.459 **	.223 *	.506 **	.431 **
humanistic practice	.362 **	.306 **	.241 **	.381 **	.327 **
**Selected SpREUK-P items**					
praying	.432 **	.348 **	.670 **	.528 **	.516 **
church attendance	.290 **	.145	.473 **	.425 **	.324 **
meditation	.452 **	.378 **	.137	.421 **	.374 **
make an effort for others	.160	.167	-.077	.065	-.006

^1^engagement in SpR practice was measured with an additional manual of the SpREUK questionnaire, the SpREUK-P manual (Büssing *et al*., 2005).

Bivariate correlations are statistically significant with ** p < 0.01; * p < 0.05 (2-tailed significance)

In detail (Table 4), praying and church attendance were strongly correlated with "Trust in external guidance" and both "Support through SpR" scales 4 and 5, while church attendance did not correlate at all with "Message of disease" and only weakly with "Search for meaningful support". In agreement with the results of our study, meditation did not correlate with "Trust in external guidance". However, an attitude of "making and effort for others" did not correlate at all with our SpREUK sub-scales.

### Analyses of variance

Next we tested the impact of several variables on the SpREUK sub-scales, such as sex and marital status, educational level and confession, age and SpR attitude, and disease and duration of disease. Using the method of univariate analyses of variance we identified several sources of variability (Table 5):

**Table 5:** Univariate variance analyses

	**Variables**	**F-value**	**significance**
**(1) Search for meaningful support**	SpR attitude age	46.429 0.784	0.000 n.s.
	Confession educational level	0.205 4.205	n.s. 0.007
	disease duration of disease	3.784 0.991	0.011 n.s.
**(2) Positive interpretation of disease**	SpR attitude age	7.710 1.128	0.000 n.s
**(3) Trust in external guidance**	SpR attitude age	82.148 1.717	0.000 0.007
	Confession educational level Confession * education	9.117 3.630 3.822	0.000 0.015 0.006
**(4) Support in relations with the External life through SpR**	SpR attitude age	51.319 1.120	0.000 n.s
	Confession educational level	0.511 2.697	n.s 0.049
**(5) Support of the Internal life through SpR**	SpR attitude age	51.319 1.120	0.000 n.s

In this table, only significant results were given. Levene's test for equality of variances was significant and thus the level of significance should be p < 0.01.

• The **SpR attitude **is an important covariate for the "Search for meaningful support", "Positive interpretation of disease", "Trust in external guidance", and both "Support through SpR" sub-scales.

• The **educational level **is an important covariate for "Search for meaningful support", "Trust in external guidance" and to a minor content for "Support in relations with the External life through SpR" – but not for the "Positive interpretation of disease".

• **Age **is an important covariate only for "Trust in external guidance".

• **Confession **is an important covariate for "Trust in external guidance".

• **Disease **itself has an impact on the "Search for meaningful support

## Discussion

Data from the current analysis demonstrate the reliability and validity of the SpREUK construct. Moreover, the sub-scales 1 and A (= 4) and B (= 5) of the preliminary version 1.0 were confirmed in the new version 1.1. In order to more precisely differentiate the three topics guidance, control and message of disease from the SpREUK version 1.0, six new items were added. Due to this fact, some items from the original item pool decreased the reliability of the construct and thus, two items from the sub-scale 1 had to be deleted ("I do not need spiritual advice" and "Spiritual/religious ideas are out-of-date", and four items which deal with the "internal/external locus of control" topic as described by Rotter [40] and Levenson [41] ("I know by myself what should be done"; "Whatever happens, I have trust in my inner strength"; "I have no influence on my life, it is fixed by fate"; "I accept my illness and bear it calmly"; "My doctor or therapist helps me to keep my illness at bay"). However, the current item pool 1 made up the new sub-scale 2 which highlights the positive interpretation of disease ("message of disease") and the new sub-scale 3 which asks for the trust in an external guidance ("God"). To improve the quality of this 3-item-scale, we have added two additional items.

The search for "meaning in life" as described by Emmons [32,33] respectively the concept of "meaning-based coping" are important topics of our questionnaire. However, the item "looking for purpose and meaning in life" loads to the sub-scale 3 which is obviously not identical with the "Search for meaningful support" through spirituality as measured in sub-scale 1. The items of sub-scale 3 fit well to the concept of "external locus of control" and share several topic with Belschner's scale "Transpersonal Trust" [43,44], while the items which made up the new sub-scale 2 (which addresses the "message of disease" and how the patients actively respond to their illness) may fit to the concept of "internal locus of control". This topic of "meaning of disease" is of outstanding importance for cancer patients [45-49], in as much as health can be conceptualized as a competence to gain control for the design of the biography [34]. In consequence, loss of control due to a life-threatening illness might be interpreted by patients as "punishment", "weakness" or "irreparable loss" – illness has no positive meaning, no "signal" to change aspects of life. As reported by Degner *et al*. [46], women who ascribed a negative meaning of illness had significantly higher levels of depression and anxiety and poorer quality of life than women who indicated a more positive meaning. As Spiritual Well-Being can be described as a 2-factor construct, i.e. Religious Well-Being and Existential Well-Being [48], addressing existentialistic concerns and the possibility to find some kind of sense and meaning even in illness are thus functions of spiritual well-being. In breast cancer patients, Levine and Tarq [8] found significant correlations of spirituality and spiritual well-being with functional well-being, while items pertaining to meaning and peace tended to correlate significantly with physical well-being. Moreover, the spirituality scales accounted for 40% of the variance in functional well-being, thus confirming the importance of spirituality and spiritual well-being in both physical and functional well-being of cancer patients.

## Conclusions

The SpREUK questionnaire may have important strengths. First, it appears to be a good choice for assessing a patients interest in spiritual concerns which is not biased for or against a particular religious commitment. Moreover, as several patients may be offended by institutional religion, even terms such as God, Jesus, praying, church etc. were avoided. Moreover, the subscale "Search for meaningful support" thus had a good correlation with both, an engagement in conventional religious practice and unconventional spiritual practice.

A second strength is that the subscale "positive reinterpretation ("message") of disease" has a good correlation with an existentialistic practice, which seems to be of outstanding importance for patients with life-changing diseases. It may be desirable to use such a measure that allows to assess attitudes which are independent of any religion or specific belief.

A third strength is that the validation was performed in a sample with at least two different types of life-changing diseases (cancer and MS, and other chronic diseases) and a healthy control group.

Beyond conceptual boundaries, our instrument differentiates the self-addressed "religious" and "spiritual" attitudes of the patients with life-threatening diseases and heeds their search for support and meaning, and integrates the topic of "meaning in illness". We cannot exclude the possibility that these topics are not relevant for healthy individuals.

In future studies we have to correlate our scales with other relevant instruments which measure aspects of SpR. Nevertheless, evaluation of the SpREUK questionnaire indicates that it is a reliable, valid measure of distinct topics of SpR that may be especially useful of assessing the role of non-religious spirituality in health related research. The focus of a larger study is to enrol patients from the highly secular Eastern Europe, and to run longitudinal studies with cancer, multiple sclerosis patients, but also cardiac failure and spinal cord damage.

The SpREUK with its additional SpREUK-P manual to measure a patient's engagement in distinct forms of SpR practice is currently available in English and German language.

## Authors' contributions

AB conceived the study, designed and developed the questionnaire, performed statistical analysis and drafted the manuscript. TO participated to conceive and design the study, performed additional statistical analysis and helped to draft the manuscript. PFM conceived the study and participated in the design and development of the questionnaire. All authors read and approved the final manuscript.
